# Enhancing Security of Telemedicine Data: A Multi-Scroll Chaotic System for ECG Signal Encryption and RF Transmission

**DOI:** 10.3390/e26090787

**Published:** 2024-09-14

**Authors:** José Ricardo Cárdenas-Valdez, Ramón Ramírez-Villalobos, Catherine Ramirez-Ubieta, Everardo Inzunza-Gonzalez

**Affiliations:** 1 Instituto Tecnológico de Tijuana, Tecnológico Nacional de México, Tijuana 22435, Baja California, Mexico; jose.cardenas@tectijuana.edu.mx (J.R.C.-V.); ramon.ramirez@tectijuana.edu.mx (R.R.-V.); catherine.ramirez193@tectijuana.edu.mx (C.R.-U.); 2 Facultad de Ingeniería Arquitectura y Diseño, Universidad Autónoma de Baja California, Carret. Tijuana-Ensenada No. 3917, Ensenada 22860, Baja California, Mexico

**Keywords:** chaotic system, multimedia data encryption, ECG, E-healthcare, H-IoT, IoT, IoMT network, RF, signal encryption, telemedicine

## Abstract

Protecting sensitive patient data, such as electrocardiogram (ECG) signals, during RF wireless transmission is essential due to the increasing demand for secure telemedicine communications. This paper presents an innovative chaotic-based encryption system designed to enhance the security and integrity of telemedicine data transmission. The proposed system utilizes a multi-scroll chaotic system for ECG signal encryption based on master–slave synchronization. The ECG signal is encrypted by a master system and securely transmitted to a remote location, where it is decrypted by a slave system using an extended state observer. Synchronization between the master and slave is achieved through the Lyapunov criteria, which ensures system stability. The system also supports Orthogonal Frequency Division Multiplexing (OFDM) and adaptive n-quadrature amplitude modulation (n-QAM) schemes to optimize signal discretization. Experimental validations with a custom transceiver scheme confirmed the system’s effectiveness in preventing channel overlap during 2.5 GHz transmissions. Additionally, a commercial RF Power Amplifier (RF-PA) for LTE applications and a development board were integrated to monitor transmission quality. The proposed encryption system ensures robust and efficient RF transmission of ECG data, addressing critical challenges in the wireless communication of sensitive medical information. This approach demonstrates the potential for broader applications in modern telemedicine environments, providing a reliable and efficient solution for the secure transmission of healthcare data.

## 1. Introduction

Cardiovascular disease (CVD) is the leading cause of death worldwide, and it is particularly associated with systolic and diastolic myocardial dysfunction, which is generally associated with atherosclerosis [1,2]. The post-pandemic effect of COVID-19, involving cardiac complications in patients who have recovered from the coronavirus, is also a factor [3,4]. Coronary heart disease, heart failure, arrhythmia, heart valve issues, and high blood pressure are among the various types of CVD [5]. Therefore, the medical and scientific community has become more engaged with the study, acquisition, and management of electrocardiogram (ECG) data [6]. Telemedicine is a wireless and remote information processing system that provides health services. Its applications are diverse and vary from the sensing of patients with diagnosed diabetes, the measurement of heart pressure, remote consultations, and the transmission of ECG and electromyography (EMG) signals for analysis and diagnosis; special initiatives have been conducted to classify and explore ECG signals and detect arrhythmias using clinical wearable atrial fibrillation (AF) recordings [7]. Additionally, studies have been carried out on different stages of sleep, analyzing RR intervals (time intervals between consecutive R-waves in the ECG signal) affected by cardiovascular arrhythmias, although they indicated only modest increases after including entropy measures [8]. This situation increased exponentially with the lack of specialists in urban areas as a direct consequence of the post-pandemic era. In Ref. [9], a multi-scale distribution entropy method is proposed to analyze the complexity of short-term heart rate variability from ECG signals, which was validated through comprehensive comparisons with traditional methods to demonstrate enhanced stability and reliability. As a result, developing algorithms for biomedical signal processing became essential.

The adoption of telemedicine systems to facilitate communication between patients and doctors to reduce the risk of infection was drastically accelerated by the COVID-19 disease [10]. At present, telemedicine is regarded by numerous researchers as a field that bridges the gap between technology and medicine; security measures must be implemented in combination with data transmission algorithms to ensure the confidentiality and integrity of patient information [11]. Furthermore, it is distinguished by a novel paradigm of E-healthcare centered around remote services [12,13]. The security of biomedical information (healthcare data) management is a current challenge in IoMT networks [14], featuring the measurement of body parameters [15,16,17], biosensors and medical images [18,19] from any Healthcare Internet of Things (H-IoT) device connected to the Internet; wireless information management systems must have adequate performance. For instance, in Ref. [20], a biometric key generation algorithm is developed for the ECG feature, and additional works show the detection of atrial fibrillation to demonstrate undiagnosed arrhythmias and prevention methods [21]. On the other hand, an encryption approach was proposed in Ref. [22] to protect ECG bundles for time-critical telecardiology applications. With the development of 5G worldwide and the need for high transmission rates and low energy consumption applications, radio-frequency (RF) transmission systems require adaptive digital modulation schemes before the digital multiplexing process. A variety of factors condition the current advancement of 5G technology. Therefore, the necessity for systems that retain complete control over channel impairments, bandwidth, power management, and bit recovery is of crucial significance, in particular, OFDM-type multiplexes with reduced bandwidths between carrier frequencies [23,24,25].

On the other hand, a dynamical system is considered chaotic when its evolution is sensitive to its initial conditions. This implies that even small initial conditions variations can yield widely diverging outcomes for such dynamical systems [26]. In this context, chaotic systems provide specific attributes that make them suitable for use as encryption systems. Data transmission security is a crucial metric for evaluating communication scheme performance. A chaotic synchronization system can enhance the reliability of secure communication schemes by providing specific properties [27]. In this particular instance, synchronization refers to the process by which two systems converge to the same dynamic. The master–slave strategy is the most commonly employed method of achieving the previously mentioned objective. In this scenario, the system exhibiting chaotic dynamics is referred to as the master system, while the system that is forced to track the dynamics of the master system is known as the slave [28].

Recently, extensive research has been conducted on master–slave synchronization within this framework to enhance the security of ECG signal transmission in telemedicine. For instance, in Ref. [29], the authors propose a novel encryption technique that utilizes signals generated in a chaotic system to encrypt information. The encrypted data can then be decrypted using a synchronization strategy incorporating the signals and a PD controller. The Lü system, a variant of the Lorenz oscillator, is employed. It consists of two chaotic systems arranged in a master–slave configuration that requires a PD controller synchronization. On the other hand, in Ref. [17], a secure communication system has been implemented using two embedded Chen chaotic systems, which are referred to as the master and slave systems, respectively. The ECG signal is encrypted using a master chaotic system. The original data are decrypted by using the slave system. To simplify the calculation of synchronization control design, the Takagi–Sugeno fuzzy method was employed to transform the master and slave components into a fuzzy format. A disturbance observer was developed to estimate the undesired signal present on public channels and the variations in parameters on both the master and slave sides. In addition, an adaptive sliding mode control was developed in combination with the disturbance observer to achieve synchronization between the master and slave systems. Both references demonstrate that synchronization errors are not exactly zero but remain close to zero. Nevertheless, this is important to the development of robust applications in chaotic systems [30,31]. In furtherance of the perspective on *feedback* methods discussed in the references given above, the synchronization problem is also closely related to the observer problem from control theory [32].

This paper aims to enhance the security and integrity of ECG data during RF wireless transmission for telemedicine applications by implementing a chaotic encryption strategy that relies on master–slave synchronization. This approach combines a multi-scroll chaotic system as the master system, with an extended state observer (ESO) acting as a slave system to estimate all states of the master, as well as the nonlinearity that generates the multi-scroll attractor. The ESO is a highly effective method that can estimate both the state vector and uncertainties in dynamical systems simultaneously [33]. In this proposal, the nonlinear term is seen as an uncertainty. Thus, in an encryption scheme, the user can modify the nonlinear term as desired, while the ESO can estimate the nonlinearity without knowing this adjustment. This feature enhances the reliability of the chaotic synchronization strategy by providing robustness. The Lyapunov stability analysis provides sufficient criteria to guarantee the boundedness of error synchronization dynamics. The proposed system supports OFDM and adaptive n-QAM schemes to optimize signal discretization. Experimentally validated, this approach prevents channel overlap during 2.5 GHz transmissions, ensuring robust and efficient data transmission in modern telemedicine environments.

The structure of this article is organized as follows: Section 2 describes the synchronization design and dynamic chaos-based random key generator algorithm and decryption stage. The processing data, n-QAM algorithm, and RF transmission algorithms are all detailed in Section 2.2. Section 3 describes the main results obtained and the spectral and performance evaluations. Finally, Section 4 shows the conclusions obtained and future work.

## 2. Proposed Scheme and RF Transmission Algorithm

### 2.1. Chaos Synchronization Design

The present research focused on utilizing a chaotic system to encrypt ECG signals, thus enhancing security in telemedicine data transmission. This proposal is based on the master–slave scheme. Firstly, as a master system, consider the following general form of a multi-scroll chaotic system, which can be described as follows:

(1a) $$\begin{array}{cc} {\dot{x}}_{1m} & =x_{2m}, \end{array}$$

(1b) $$\begin{array}{cc} {\dot{x}}_{2m} & =x_{3m}, \end{array}$$

(1c) $$\begin{array}{cc} \;{\dot{x}}_{3m} & =-a_{1}x_{1m}-a_{2}x_{2m}-a_{3}x_{3m}+f\left(x_{1m},x_{2m},x_{3m}\right), \end{array}$$

where $\{a_{1},\;a_{2},\;a_{3}\}\in R$, and f(·) is a nonlinear function that generates 1-directional multi-scroll chaotic attractors, and it may be defined by several topologies (e.g., hyperbolic functions, piecewise linear function (PWL), special form of a sine function (SFSF)) [34].

Further, we consider the nonlinear function f(·) as an additional state, i.e., ${\dot{x}}_{4m}=\varphi \left(t\right)$. Consequently, system (1) can be represented by the following extended state system:

(2a) $$\begin{array}{cc} {\dot{x}}_{1m} & =x_{2m}, \end{array}$$

(2b) $$\begin{array}{cc} {\dot{x}}_{2m} & =x_{3m}, \end{array}$$

(2c) $$\begin{array}{cc} \;{\dot{x}}_{3m} & =-a_{1}x_{1m}-a_{2}x_{2m}-a_{3}x_{3m}+x_{4m}, \end{array}$$

(2d) $$\begin{array}{cc} \;{\dot{x}}_{4m} & =\varphi (t). \end{array}$$

Secondly, the slave system is described by the following ESO:   

(3a) $$\begin{array}{cc} \;{\dot{x}}_{1s} & =x_{2s}+l_{1}\left(y-x_{1s}\right) \end{array}$$

(3b) $$\begin{array}{cc} \;{\dot{x}}_{2s} & =x_{3s}+l_{2}\left(y-x_{1s}\right) \end{array}$$

(3c) $$\begin{array}{cc} \;{\dot{x}}_{3s} & =-a_{1}x_{1s}-a_{2}x_{2s}-a_{3}x_{3s}+x_{4s}+l_{3}\left(y-x_{1s}\right) \end{array}$$

(3d) $$\begin{array}{cc} {\dot{x}}_{4s} & =l_{4}\left(y-x_{1s}\right) \end{array}$$

where $y=x_{1m}$ denotes the measurable output of (1), and $l_{i}\;\left(i=1,2,3,4.\right)$ represents the observer gains. In this approach, the estimation of all states, including the function f(·), is performed via the ESO.

To address the synchronization problem between (1) and (3), we define the error state as $e_{i}\left(t\right)=x_{im}-x_{is},\;\left(i=1,2,3,4\right)$. Thus, the error dynamic system is described as follows:

(4a) $$\begin{array}{cc} {\dot{e}}_{1} & =-l_{1}e_{1}+e_{2}, \end{array}$$

(4b) $$\begin{array}{cc} {\dot{e}}_{2} & =-l_{2}e_{1}+e_{3}, \end{array}$$

(4c) $$\begin{array}{cc} \;{\dot{e}}_{3} & =-\left(a_{1}+l_{3}\right)e_{1}-a_{2}e_{2}-a_{3}e_{3}+e_{4}, \end{array}$$

(4d) $$\begin{array}{cc} {\dot{e}}_{4} & =\varphi \left(t\right)-l_{4}e_{1}. \end{array}$$

The *main objective* is to present a design technique that can deal with variations in the nonlinear function $f(x_{1m},x_{2m},x_{3m})$ in system (1) and solve the problem of chaotic synchronization. Here, we analyze the stability of system (4) through Lyapunov criteria. First, let us define the vector error state $e≜{\left[e_{1},\ldots ,e_{4}\right]}^{T}$. Therefore, the error dynamic (4) can be expressed as follows:

(5) $$\dot{e}=A_{e}e+B\varphi \left(t\right),$$

with

(6) Ae≜−l1−1−0−0−l2−0−1−0−a1−l3−a2−a3−1−l4−0−0−0,B≜0001.

Here, we can select the values of observer gains $l_{i}$ such that all eigenvalues of matrix $A_{e}$ have a negative real component. Meanwhile, the following is now considered to hold from this point forward:

**(A1)**  The solutions of system (1) remain uniformly bounded for all time t∈[0,∞).
**(A2)**  The scalar function φ(t) in (5) is an unknown function with *a priori* known upper bound, denoted as $\varphi^{+}>0$, such that
  (7) $$|\varphi \left(t\right)|\leq \varphi^{+}.$$

Now, for the stability analysis of system (5), consider a Lyapunov function defined as $V\left(e\right)=e^{T}Pe$. Here, $P\in R^{4\times 4}$ is a positive definite and symmetric matrix. The time derivative of $\dot{V}\left(e\right)$ along the trajectories of (5) yields the following:

(8) $$\dot{V}\left(e\right)=e^{T}\left(PA_{e}+A_{e}^{T}P\right)e+2e^{T}PB\varphi \left(t\right).$$

If we choose a solution *P* that satisfies the following Lyapunov equation,

(9) $$PA_{e}+A_{e}^{T}P=-Q,$$

for any positive definite matrix *Q*, subsequently, $\dot{V}\left(e\right)$ can be stated as follows:

(10) $$\dot{V}\left(e\right)=-e^{T}Qe+2e^{T}PB\varphi \left(t\right).$$

Then, based on Assumption (A2), the function $\dot{V}\left(e\right)$ is reformulated as follows:

(11) $$\dot{V}\left(e\right)\leq -(\lambda_{\min}\left\{Q\right\}∥e∥-2\varphi^{+}\lambda_{\max}\left\{P\right\})∥e∥.$$

Here, $\dot{V}\left(e\right)$ is a negative definite function for all e(t) outside the set

(12) $$B=\left\{e:∥e∥>\frac{2\varphi^{+}\lambda_{\max}\left\{P\right\}}{\lambda_{\min}\left\{Q\right\}}\right\}.$$

Thus, the trajectories of the error state dynamic (5) are uniformly bounded. As a result, the solution of the slave system (3) will be bounded around the solutions of the master system (1).

The proposed algorithm has an important benefit in that it does not require knowledge of function $f(x_{1},x_{2},x_{3})$ of the master system (1). This feature makes it especially desirable for synchronizing chaotic systems. In addition, similar to the instance of Refs. [35,36], the observer gains may be chosen such that they minimize the estimation error.

*Example.* To illustrate the effectiveness of the proposed scheme, let us consider the following multi-scroll chaotic system introduced in Ref. [37] as the master system:

(13a) $$\begin{array}{cc} {\dot{x}}_{1m} & =10x_{2m}, \end{array}$$

(13b) $$\begin{array}{cc} {\dot{x}}_{2m} & =10x_{3m}, \end{array}$$

(13c) $$\begin{array}{cc} \;{\dot{x}}_{3m} & =10\left(-ax_{2m}-cx_{3m}-d_{1}f\left(x_{1m}\right)\right). \end{array}$$

with

(14) f(x1m)=+sin(2πbx1m),−knb>x1m,−sin(2πbx1m),−knb≤x1m≤kpb,+sin(2πbx1m),−kpb<x1m,

where $\{a,\;b,\;c,\;d_{1}\}\in R$ and $\left\{k_{n},\;k_{p}\right\}\in N^{+}$. In system (13), the scroll numbers and the size of the attractor can be arbitrarily modified by a tuning of the parameters in function $f(x_{1m})$. The system (13) has the following characteristics:

(i) It can present a chaotic, cyclical or stable dynamic.
(ii) By tuning the parameters $k_{n}$ and $k_{p}$, generating a chaotic attractor with several scrolls is possible. The number of scrolls can be distributed on the right and left side of the phase plane $\left(x_{1m},x_{2m}\right)$.

For study purposes, we only concentrated on the chaotic behavior of system (13). Now, the nonlinear function (14) is considered as an additional state of (13). Thus, the master system can be described as the following extended state system:

(15a) $$\begin{array}{cc} {\dot{x}}_{1m} & =10x_{2m}, \end{array}$$

(15b) $$\begin{array}{cc} {\dot{x}}_{2m} & =10x_{3m}, \end{array}$$

(15c) $$\begin{array}{cc} \;{\dot{x}}_{3m} & =10(-ax_{2m}-cx_{3m}+x_{4m}), \end{array}$$

(15d) $$\begin{array}{cc} {\dot{x}}_{4m} & =10\varphi (t), \end{array}$$

(15e) $$\begin{array}{cc} y & =x_{1m}, \end{array}$$

where φ(t) is an unknown and bounded function. Meanwhile, the slave system is designed as follows:

(16a) $$\begin{array}{cc} {\dot{x}}_{1s} & =10\left(x_{2s}+l_{1}\left(y-x_{1s}\right)\right), \end{array}$$

(16b) $$\begin{array}{cc} {\dot{x}}_{2s} & =10\left(x_{3s}+l_{2}\left(y-x_{1s}\right)\right), \end{array}$$

(16c) $$\begin{array}{cc} \;{\dot{x}}_{3s} & =10\left(-ax_{2s}-cx_{3s}+x_{4s}+l_{3}\left(y-x_{1s}\right)\right), \end{array}$$

(16d) $$\begin{array}{cc} \;\;{\dot{x}}_{4s} & =10\left(\varphi \left(t\right)+l_{4}\left(y-x_{1s}\right)\right). \end{array}$$

In the simulation, the implicit Euler method (with a step size $h=1\times 10^{-3}$) is used to solve the system (13) and its corresponding slave system (16). The parameter values for the master system are $a=0.3,\;b=0.25,\;c=0.3,\;d=0.35,\;k_{n}=3,\;k_{p}=2$, and the observer gains $l_{i}$ were tuned using the pole placement approach with $l_{1}=\frac{2}{5}\omega_{0}-c,\;l_{2}=\frac{3}{50}\omega_{0}^{2}-cl_{1},\;l_{3}=\frac{2}{500}\omega_{0}^{3}-a-cl_{2},\;l_{4}=\frac{1}{10000}\omega_{0}^{4}$ for $\omega_{0}=50$ rad/sec. The initial conditions of the master system (13) and the slave system (16) are set as

(17a) $$\begin{array}{cc} \;{\left[\begin{array}{ccc} x_{1m}\left(0\right), & x_{2m}\left(0\right), & x_{3m}\left(0\right) \end{array}\right]}^{T}\; & =0.1\in R^{3}, \end{array}$$

(17b) $$\begin{array}{cc} {\left[\begin{array}{cccc} x_{1s}\left(0\right), & x_{2s}\left(0\right), & x_{3s}\left(0\right), & x_{4s}\left(0\right) \end{array}\right]}^{T}\; & =0.5\in R^{4}. \end{array}$$

The largest Lyapunov exponent, denoted as $\lambda_{LE}$, is equal to 0.1092 for the set of parameter values. A positive value of $\lambda_{LE}$ indicates that the system described by (13) exhibits chaotic behavior.

**Figure 1:**
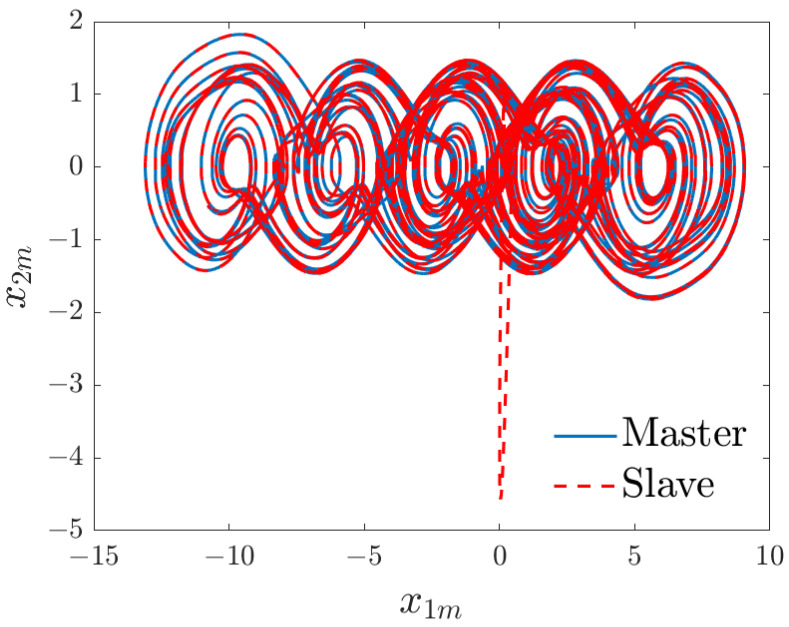
Chaotic attractor.

**Figure 2:**
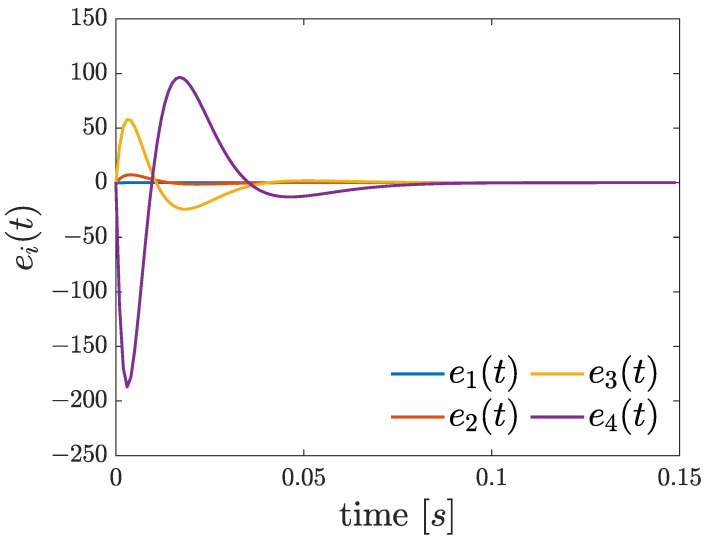
Error state responses.

The simulation results are illustrated in Figure 1 and Figure 2. The trajectories of the slave system converge toward the chaotic attractor of the master system (see Figure 1). In addition, as depicted in Figure 2, the error states converge to zero and remain around its vicinity.

### 2.2. Chaotic Encryption and n-QAM

Algorithm 1 performs the chaotic encryption by executing an additive combination of the biomedical signal (ECG) with the chaotic signal (cS) from the master system (13) [38,39]. The purpose of this additive combination is to mask the original ECG data by allowing the chaotic signal to dominate, making the original signal unrecognizable and thus encrypted. In this stage, the encryption process itself does not add complexity; it arises during the modulation stage.
**Algorithm 1** Modulation and Encryption of ECG Signal**Input** Biomedical signal ECG, Chaotic signal cS, Modulation order *M* **Output** Modulated signal mS
  1:**procedure** Preprocess_Encrypt_Signal  2:    **def** M=n-QAM  3:    eS←ECG+cS                                                                                                             ▹ ECG signal combined with chaotic signal  4:    sN←[]                                                                        ▹ Initialize the normalized signal array according to the n-QAM order  5:    **for** each $eS_{i}$ in eS **do**  6:        $sN\leftarrow \frac{eS_{i}-min\left(\mathrm{eS}\right)}{max(\mathrm{eS})-min(\mathrm{eS})}$  7:    **end for**  8:    **return** sN  9:    sD←[]                                                                                                                               ▹ Initialize the discretized signal array10:   **for** each $sN_{i}$ in sN **do**11:        $\mathrm{sD}\leftarrow \mathrm{floor}(sN_{i}\cdot {\left(M-1\right)}^{2})$                                                                      ▹ Signal rounding and conversion to complex signal12:    **end for**13:    **return** sD14:    ECG_ds ← {real(sD), imag(sD)}                                                                                                    ▹ Decomposition of the signal15:    mS←n-QAM(ECG_ds,M)                                               ▹ Modulation of the encrypted signal based on the n-QAM order16:    **return** mS17:**end procedure**


Before modulation, each value of the encrypted signal is normalized by subtracting its minimum value and dividing by the difference between the maximum and minimum values, yielding the normalized signal (sN). The proposed Algorithm 1 continues determining the discrete value for each normalized value that falls within the range of values of the modulation order *M*. The n-QAM technique then modulates the signal using two channels: I (in-phase) and Q (quadrature). The “n” represents the number of symbols corresponding to specific amplitude and phase combinations. The process begins with digitizing the normalized signal (sN) into a serial bit-stream, which is then split into phase shift and amplitude change stages. This creates a new signal (sD) with a 2.45 GHz carrier frequency. The transmitted signal is complex due to these variations, as each transmitted symbol is represented by a vector sent by RF and is graphed as an n-QAM constellation; this is depicted in the complex variable ECG_ds. The final output of the proposed algorithm is a complex modulated signal (mS) prepared for secure transmission.

### 2.3. RF Transmission Algorithm

The procedure for RF transmission is outlined in Algorithm 2. Implementing a long-term evolution (LTE) System Toolbox, the algorithm replicates an LTE signal that satisfies the requirements of a realistic signal transmission environment. The process starts by setting the configuration parameters for the transmission based on the needs of the transmitting signal. These parameters are the type of configuration, sampling rate, bandwidth and filter. Using these parameters, the proposed algorithm generates the reference measurement channel (RMC) waveforms. The algorithm also implements a root-raised cosine type filter ($ECG_{TX}$) before transmitting the signal to reduce inter-symbol interference, improving the quality and reliability of the signal. The AD9361 transceiver is initiated by setting up the input (inputTX) and output (outputRX) channels of the card once the signal is prepared. Finally, the proposed algorithm transmits the encrypted signal on the 2.45 GHz frequency and receives it on the RX1 channel ($ECG_{RX}$). As an overview, the transmission methodology is described as follows:

1. Declare the specific parameters for LTE signal configuration.
2. Generate the RMC waveform following the established parameters.
3. Implement a root-raised cosine filter and signal strength adjustment.
4. Define transmission and reception parameters for the AD9361.
5. Transmit the generated signal through the set transmitter.
6. Use of the configured receiver to capture the transmitted signal.

**Algorithm 2** Generation and Transmission of LTE Signal**Input** Modulated signal mS **Output** Received signal $ECG_{RX}$
  1:**procedure** Generate_Transmit_LTE_Signal  2:    **LTE Parameters Configuration**  3:    Set LTE configuration parameters  4:    Generate RMC signal with specified parameters state  5:    **Transmission**  6:    $ECG_{TX}\leftarrow$ filter_design(mS, rolloff, span)  7:    inputTX ← {real($ECG_{TX}$), imag($ECG_{TX}$)}  8:    AD9361_TX(inputTX) ▹ Setup AD9361 for signal transmission.  9:    AD9361_RX(outputRX)    ▹ Setup AD9361 for signal reception.10:   Signal transmission11:   $ECG_{RX}\leftarrow$ AD9361_RX(outputRX) ▹ Reception on channel RX112:    **return** $ECG_{RX}$13:**end procedure**


### 2.4. Signal Post-Processing and Demodulation

Signal post-processing ensures the data are aligned at the correct position in the complex plane and the data are demodulated. This procedure is detailed in Algorithm 3, which initiates with synchronizing the transmitted and received signals (RX_syn) to guarantee that both signals are in time sync. The algorithm proceeds by rotating the polar shape of the signal that has already been synchronized. RX_rot displays the formula for constellation rotation. The value θ is variable and is defined based on a visual assessment of the received constellation angles. Therefore, the value of θ is adjusted as needed in real time. The original signal’s characteristics are recovered by de-filtering the signal (RX_f) before signal demodulation. The signal is recovered into its digital data using the n-QAM function (demodS), which is then concatenated into a single variable (ECG_complex). The subsequent steps are equivalent to the inversion of the discretization and normalization procedure, in which each value of the encrypted signal is restored to its original state in time. In summary, the procedure involves the following steps:

1. Align the transmitted and received signals.
2. Rotate the synchronized signal’s polar shape by modifying θ.
3. De-filter the signal.
4. Demodulate the signal.
5. Concatenate the demodulated signal into a single variable.
6. Reverse the discretization and normalization process to return each encrypted signal value to its original state.

**Algorithm 3** Post-processing of received signal.**Input** Received signal $ECG_{RX}$, Transmitted signal $ECG_{TX}$, n-QAM order *M* **Output** Encrypted signal ECG_encrypt
  1:**procedure** Process_Received_Signal  2:    $RX\_syn\leftarrow \mathrm{synchronize}(ECG_{RX},ECG_{TX})$  3:    RX_rot←RX_syn·exp(i·θ)          ▹ Rotation in the complex plane  4:    RX_f←filter_design(RX_rot)  5:    demodS←qamdemod(RX_f,M)  6:    *ECG_complex* ←{real(demodS), imag(demodS)}  7:    ECG_d←[]  8:    **for** each $ECG\_complex_{i}$ in ECG_complex **do**  9:        $ECG\_d\leftarrow \frac{\mathrm{ECG}\_{\mathrm{complex}}_{i}}{{\left(M-1\right)}^{2}}$10:   **end for**11:   **return** ECG_d12:   ECG_encrypt←[]13:   **for** each $ECG\_d_{i}$ in ECG_d **do**14:       $ECG\_encrypt\leftarrow ECG\_d_{i}\cdot \left(\max-\min\right)+\min$15:   **end for**16:   **return** ECG_encrypt17:**end procedure**


### 2.5. Signal Decryption

Algorithm 4 is implemented during the final stage of the signal decryption process. The parameters of the master system are established, and the profits of the observer are initialized. The proposed algorithm then iterates over the synchronization signal (x1) duration, using the Euler method to approximate the state variables continuously. These variables estimate the chaotic components of the signal. The decrypted ECG signal (ECG_dec) is obtained by subtracting the approximated chaotic signal (x3s) from the encrypted signal (ECG_encrypt) that was received. The synchronization of the original and estimated signals emphasizes the observer’s accuracy. In summary, the algorithm is defined by the subsequent stages:

1. Establish the parameters of the master system and the gains of the observer.
2. Approximate the observer variables using the Euler method, defining the time step and initial variables.
3. Subtract the observer’s estimated signal from the encrypted signal to decrypt the ECG signal.

**Algorithm 4** Decryption of ECG Signal**Input** Encrypted signal ECG_encrypt, synchronization signal **Output** Decrypted ECG signal ECG_dec
  1:**procedure** Observer Synchronization Process  2:    Initialize parameters  3:    Initialize observer gains  4:    N← Length of dS  5:    Initialize states  6:    dt← Defined time step  7:    **for** i←1 to N−1 **do**  8:        x1s[i+1]←x1s[i]+h·(x2s[i]+l1·(x1[i]−x1s[i]))  9:        x2s[i+1]←x2s[i]+h·(x3s[i]+l2·(x1[i]−x1s[i]))10:        x3s[i+1]←x3s[i]+h·(−a1·x1s[i]−a2·x2s[i]−a3·x3s[i]+x4s[i]+l3·(x1[i]−x1s[i]))11:        x4s[i+1]←x4s[i]+h·4·(x1[i]−x1s[i])12:   **end for**13:   ECG_dec←ECG_encrypt−x3s14:   **return** ECG_dec15:**end procedure**


**Figure 3:**
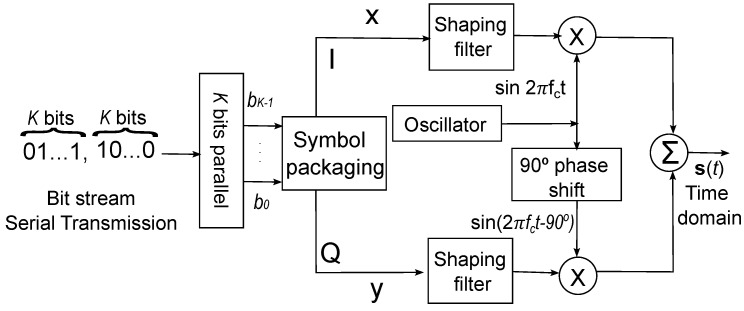
Architecture of n-QAM scheme.

An n-QAM algorithm was developed as an essential part of the transmission algorithm for the processing of ECG data. The system is adaptive for variable n-QAM resolution with the input stream consisting of $x=[x_{1},...,x_{N}]$ for the I-Channel, $y=[y_{1},...,y_{N}]$ for the Q-Channel, and a 2.45 GHz local oscillator that integrates the in-phase and quadrature components. The resulting signal vector $s\left(b\right)=[\left(x_{1},y_{1}\right),...\left(x_{N},y_{N}\right)]$ is composed of *K* bits of binary symbols. Figure 3 depicts the developed architecture of n-QAM:

The AD9361 transceiver sends the time-domain structure of the symbol packaging stage, which is based on K symbols modulated in the final waveform $s\left(t\right)=[s_{1}\left(t\right),...,s_{N}\left(t\right)]$, to the transmission chain outside the transceiver. This chain comprises a bandpass filtering stage, the power amplifier under test, and the return signal obtained through the directional coupler and spectral analysis [23].

The following equation can be used to express the general QAM equation.

(18) $$s\left(t\right)=I\left(t\right)\sin\left(2\pi f_{c}t\right)+Q\left(t\right)\sin\left(2\pi f_{c}t-90^{\circ }\right),$$

where the carrier frequency is denoted by $f_{c}$, and a coherent demodulator at the receiver multiplies the received signal with a cosine and sine signal to produce the received estimates of I(t) and Q(t). By employing conventional trigonometric identities, this can be expressed as Equation (19):

(19) $$s\left(t\right)=\frac{1}{2}I\left(t\right)+\frac{1}{2}\left(I\left(t\right)\cos\left(4\pi f_{c}t\right)-Q\left(t\right)\sin\left(4\pi f_{c}t\right)\right),$$

The representation of digital transmission denoted by the n-QAM constellation is represented as follows

A signal which is represented in an n-QAM constellation can be expressed as follows:

(20) QAM(t)=s(t)+n(t),

where n(t) represents the additive white Gaussian noise (AWGN), while s(t) denotes the signal modulated under an n-QAM scheme mainly determined by the n scheme under the transmission, which can be defined as

(21) $$s_{k}\left(t\right)=a_{j}g\left(t-nT_{B}\right)\sin\left(2\pi f_{c}t\right)+b_{j}g\left(t-nT_{B}\right)\sin\left(2\pi f_{c}t+90^{\circ }\right),$$

where $a_{j}$ and $b_{j}$ represent the in-phase and quadrature, respectively. The function g(·) is the base-band tone, and $T_{B}$ represents the symbol time duration.

### 2.6. Summarize

In summary, the design procedure for chaotic encryption and transmission can be summarized as follows:

Step 1: Construct the master–slave system stated in Equations (1) and (3).
Step 2: Apply any pole assignment technique to compute the values of observer gains $l_{i},\;\left(i=1,2,3,4\right)$ to ensure that all eigenvalues of matrix $A_{e}$ have a negative real part, i.e., $ℜ(\lambda_{i}\left\{A_{e}\right\})<0$.
Step 3: Combine the ECG signal with the chaotic signal to generate the encrypted ECG signal. Subsequently, the signal is pre-processed for modulation using n-QAM.
Step 4: Set the configuration parameters and generate the RMC waveform for LTE transmission. Subsequently, transmit the encrypted ECG signal.
Step 5: The encrypted ECG signal is post-processed through an n-QAM demodulation process and filtering stage.
Step 6: Set the configuration parameters for decryption. Subsequently, the decryption of the ECG signal is carried out; i.e., the ECG signal is reconstructed by subtracting the chaotic encryption signal from the encrypted signal.

**Figure 4:**
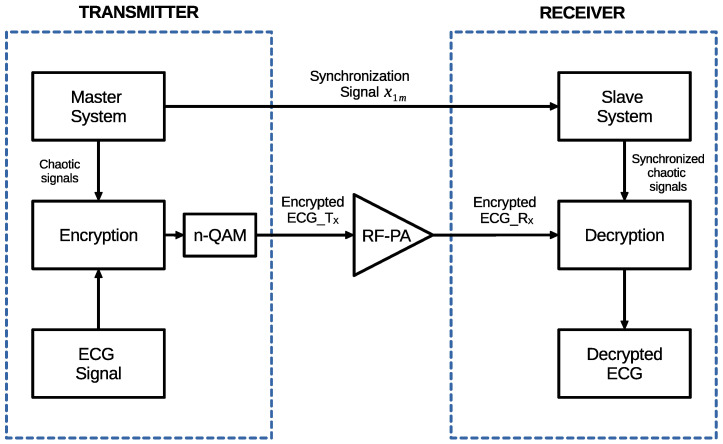
Overall diagram scheme.

An overall diagram of the proposed chaotic encryption and transmission scheme is presented in Figure 4.

## 3. Experimental Results

**Figure 5:**
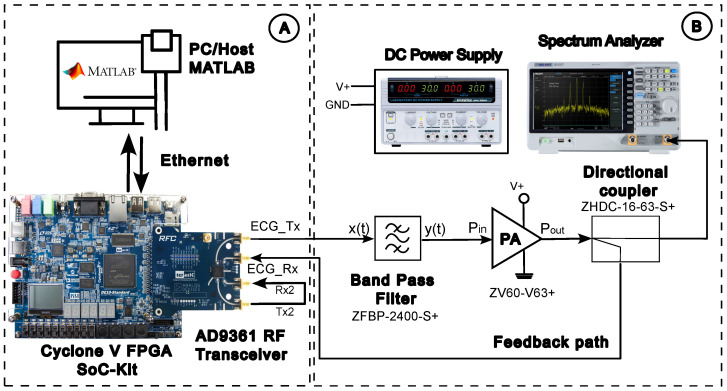
Block diagram of the transmission testbed proposed. Part A: Signal transmission and control. Part B: Signal path and measurement.

A block diagram of the experimental RF system is depicted in Figure 5. Part A proposes using a PC/Host that runs MATLAB version R2024a and is connected to a Cyclone V FPGA SoC development kit manufactured by Intel Corporation (Altera), (Santa Clara, CA, USA) via Ethernet. For the transmission process of the encrypted $ECG_{TX}$ signal, one of the channels of the dual-band transceiver is used that contains the biological ECG information of interest added to the chaotic information. In this work, the synchronization channel $x_{1s}$ is carried out in the Host. In the simulation, the decryption key is not sent through the same wireless medium operating in the same 2.45 GHz RF band. If the transmitted synchronization signal is intercepted, the purpose of guaranteeing the privacy of the information would be lost. It is essential to send by an alternative path or, even better, an embedded system located in the specialist doctor to contain the encryption key as a further implementation stage. Finally, the PC/Host regulates and configures the transmission and reception of signals by utilizing MATLAB to store the data in the FPGA’s DDR memories. Part B of the diagram corresponds to the analog proposal in which the signal transmitted by the AD9361 transceiver is subjected to the ZFBP-2400-S+ bandpass filter amplified by a ZV60-V63+ power amplifier. Lastly, a ZV60-63-S+ directional coupler is employed to divide the amplified signal, enabling the spectrum analyzer to analyze a portion of it while the remaining portion continues its transmission path. The amplifier operates on the energy supplied by the DC power supply. The feedback path enables real-time adjustments and corrections based on the analysis of the transmitted signal by allowing a portion of the amplified signal to return to the FPGA SoC-Kit.

**Figure 6:**
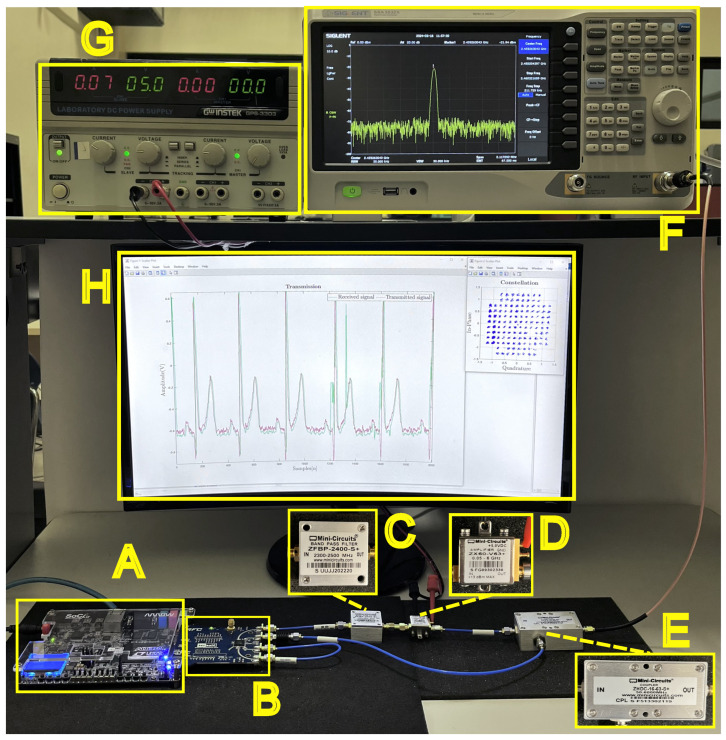
Photo of the experimental testbed. Equipment pertinent to the setup: (**A**) Altera Cyclone V FPGA SoC-Kit. (**B**) AD9361 RF Agile Transceiver operating at a center frequency of 2.45 GHz. (**C**) Mini-circuits ZFBP-2400-S+ bandpass filter. (**D**) Mini-circuits for power amplifiers ZX60-V63+. (**E**) Coupler mini-circuits ZHDC-16-63-S+. (**F**) SIGLENT SSA 3032X Spectrum Analyzer. (**G**) GW INSTEK GPS-3303 Power Supply. (**H**) Display HOST PC-MATLAB R2024a.

The experimental design considers the collection of real-world biological data. The implemented transmission must be located in the linear region of the power amplifier. The RF-PA under broadband test with high dynamic range for LTE applications has an output power of 1 dB compression of 17.8 dBm, which sends it to saturation levels, so the transmission developed is below this power threshold. Thus, implementing the model and the experimental results are appropriate for the proposed system depicted in Figure 6, which employs a software-defined radio (SDR) transceiver base. The SDR platform includes a system-on-chip (SoC) device and a software and hardware interface for developing the SDR system. For the in-phase (*I*) and quadrature (*Q*) signals, the RF conversion system uses direct conversion with 2×2 channels. The testbed configuration includes the following components: a ZFBP-2400-S+ bandpass filter with a bandwidth of 2400 MHz ± 100 MHz and an insertion loss of approximately 2 dB; a ZX60-V63+ power amplifier (with a 5 V bias) offering a gain of approximately 20 dB when operated at a frequency of 2.45 GHz; and a ZHDC-16-63-S+ coupler with a high directivity of 32 dB typ. and a return loss of approximately 23 dB. An attenuator with a 20 dB is installed at the input to protect the spectrum analyzer. A 3 dB attenuator is also positioned at the input of the RX channel of the transceiver board to protect the receiving signal below the power threshold.

The developed platform contains the following measurement equipment, an FPGA embedded system and an RF transceiver: SIGLENT SSA 3032X Spectrum Analyzer, SIGLENT’s headquarters is in Shenzhen, China. The power supply GW INSTEK GPS-3303 is manufactured by Good Will Instrument Co., whose headquarters are in Taipei, Taiwan. Cyclone V FPGA SoC-Kit is manufactured by Intel Corporation (formerly Altera), headquartered in Santa Clara, CA, USA. AD9361 RF Agile Transceiver manufactured by Terasic located in Hsinchu, Taiwan.

In relation to the analog devices for signal and power management, the following devices were used: Mini-Circuits ZFBP-2400-S+ Bandpass Filter: Mini-Circuits is based in Brooklyn, New York, USA. Mini-Circuits ZX60-V63+ Power Amplifiers: Mini-Circuits, Brooklyn, New York, NY, USA. Mini-Circuits ZHDC-16-63-S+ Coupler: Mini-Circuits in Brooklyn, New York, NY, USA.

### Decryption and Spectral Validation

In a rural sector, RF transmissions provide the advantage of transmitting information without the requirement of Wi-Fi or Ethernet. In this scenario, a line-of-sight transmission of relevant biological information would suit an embedded system like the Cyclone V FPGA development board. The transmission was devised for a carrier frequency of 2.5 GHz to be interpreted by a specialist. The root mean squared error (RMSE) metric, defined in Equation (22), determines the efficacy of the transmitted signal concerning the received ECG signal.

(22) $$RMSE=\sqrt{\sum_{n=1}^{N}{\left[x_{tx}\left(n\right)-x_{rx}\left(n\right)\right]}^{2}},$$

where $x_{tx}\left(n\right)$ is the ECG transmitted signal and $x_{rx}\left(n\right)$ is the received signal under test.

**Figure 7:**
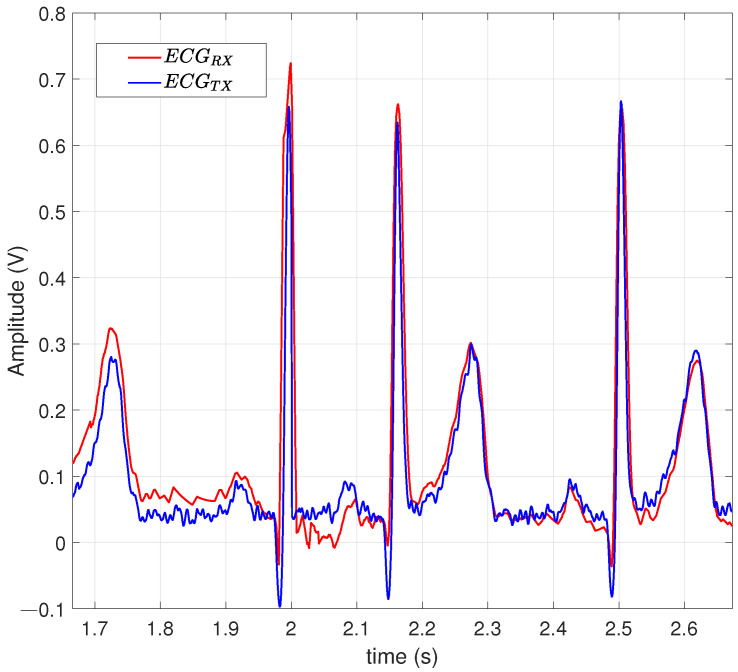
A 128-QAM with a power amplifier using a scale factor of 0.05.

Figure 7 contains a comparison between an $ECG_{Rx}$ signal and an $ECG_{Tx}$ signal at a heart rate of 1.11 Hz, with a precision of RMSE of 0.0235, using a 128-QAM modulation scheme.

**Figure 8:**
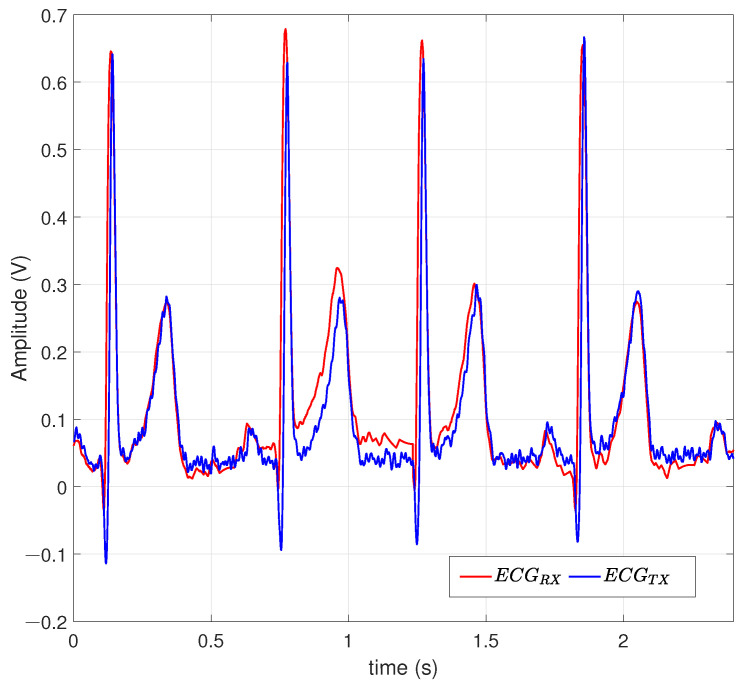
An ECG signal decrypted under a 128-QAM scheme.

Figure 8 illustrates an ECG signal of 1.66 Hz. The demonstration was carried out for a medium-resolution 128-QAM scheme that provides adequate precision for interpreting signals by a medical specialist in the field; the system is adaptable to n-QAM variations. The implementations that have been conducted achieved a precision of RMSE of 0.0180 for ECG signals. The predominant frequencies are recovered to diagnose pertinent pathologies from the signals transmitted through the RF transmission chain.

**Figure 9:**
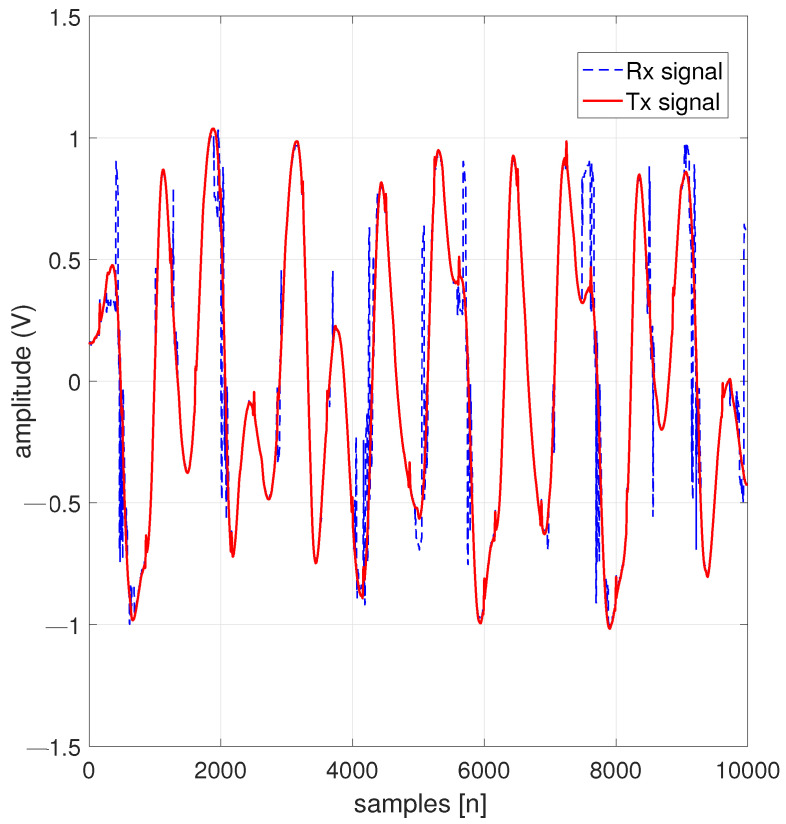
ECG signal encrypted under the 128-QAM modulation scheme.

**Figure 10:**
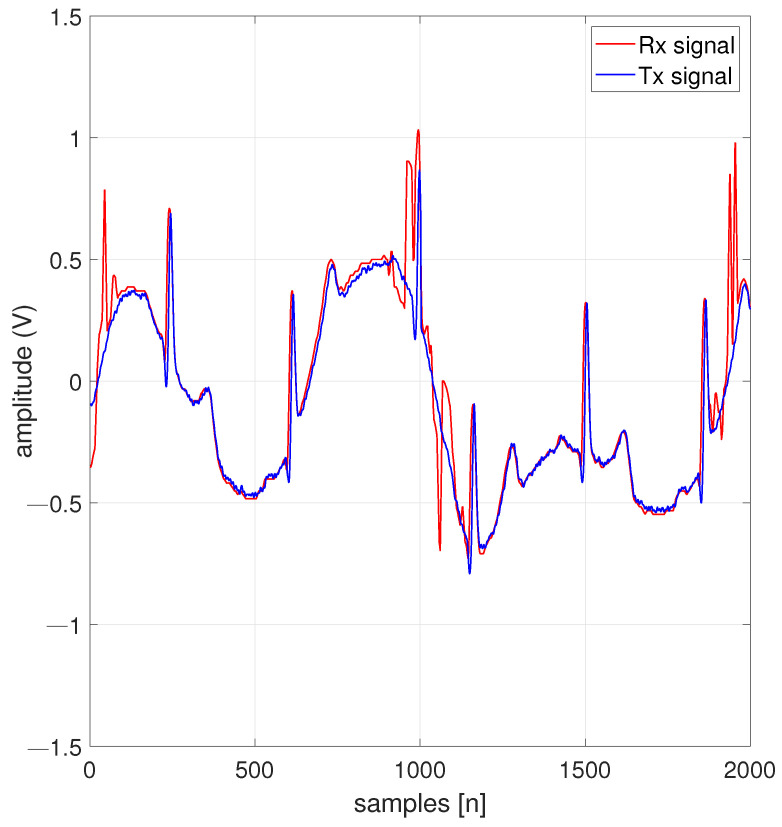
ECG signal with tachycardia encrypted under the 128-QAM modulation scheme.

The ECG signal under test is compared to the chaotic system signal implementations in Figure 9 and Figure 10. When the chaotic system is implemented, the transmitted signals are evaluated with the decrypted signals, and a precision that improves an RMSE 0.0559 is obtained. The demodulation process and the elimination of the chaotic signal significantly enhance the accuracy of the resultant ECG. The synchronization matter, which is a result of the chaotic system synchronization error, is resolved by Algorithm 1 through the modulation and encryption of the ECG signal and Algorithm 2 through the generation of the LTE signal that will be transmitted through the AD9361 transceiver.

**Figure 11:**
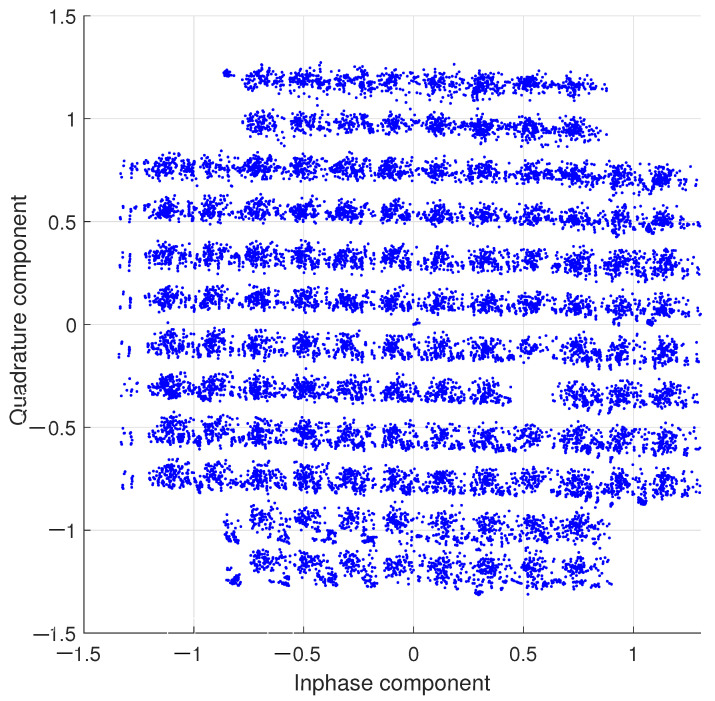
128-QAM constellation of an encrypted ECG signal.

The error vector measurement (EVM) validation of the encrypted ECG signal under a 128-QAM modulation scheme is illustrated in Figure 11. An accuracy of -27 (db) EVM is achieved in the implementation. Upgrading to a higher-order n-QAM scheme can enhance this measurement. The original signal can be recovered by decompressing the 7-bit symbols. The ECG signal, sent for a future classification and diagnosis procedure, is obtained by subtracting the chaotic signal.

**Figure 12:**
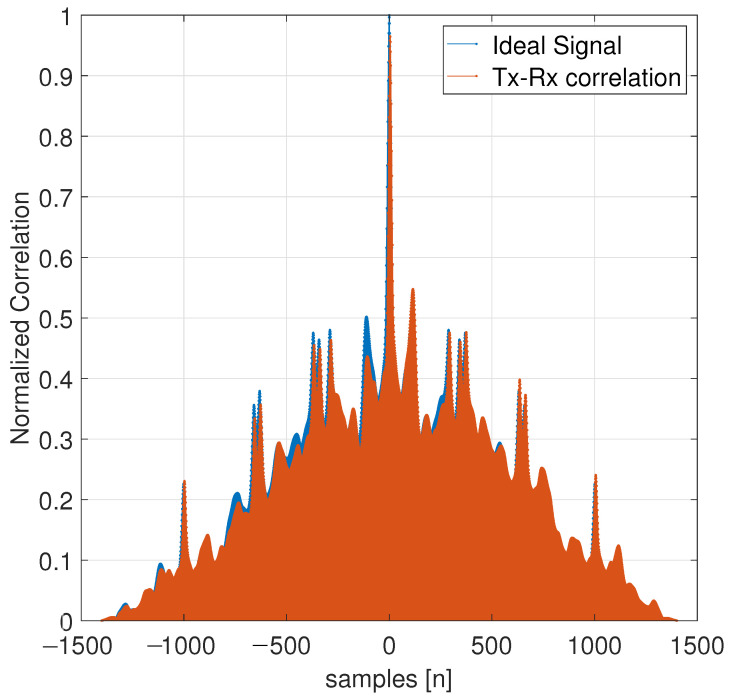
Cross-correlation of ideal received signal and transmitted–received ECG signal.

Figure 12 illustrates two comparisons of the correlation between the ideal signal (blue) and the cross-correlation of the received signal in relation to the transmitted signal (brown). The correlation between the recovered and transmitted signals is comparable, as evidenced by the maximum amplitude at the origin. Figure 8 contains four ECG cycles with a population of 1402 samples, which are broken down along Figure 12. The QRS complexes of the recovered and transmitted signals are primarily located in the ranges of 100, 400, 1300, and 2050 sample numbers.

**Figure 13:**
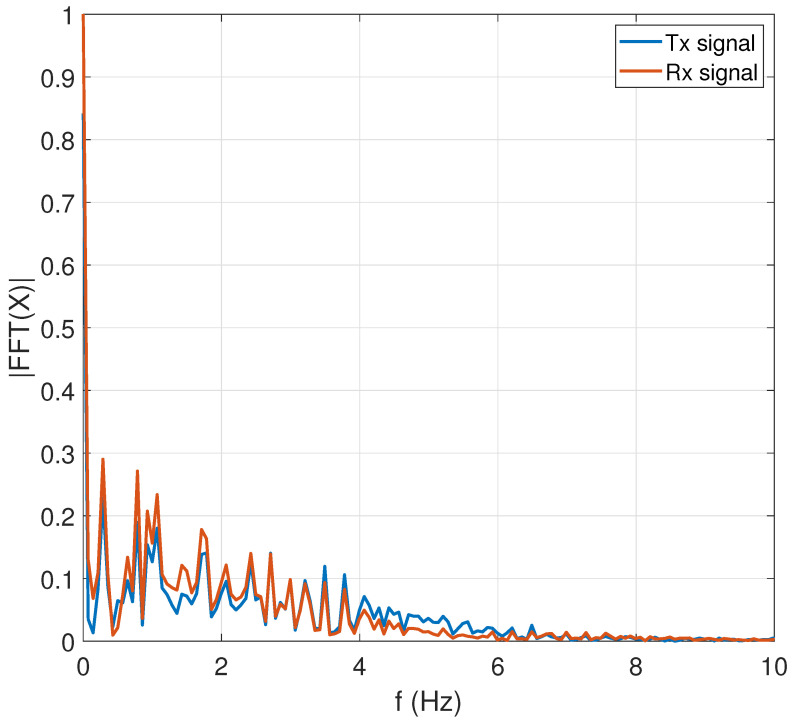
Discrete Fourier transform of transmitted and received ECG signal.

Figure 13 shows the discrete Fourier transform (DFT) of the transmitted and received signals. The signals under test oscillate at a frequency of 1.66 Hz, and with DC components as shown in the origin, the power begins to dissipate right in the range of 8 Hz, which includes the inner frequencies observed in Figure 8 in the Q-T wave at the end of each period of the ECG. Analysis in the frequency domain allows us to analyze and diagnose pathologies and arrhythmias. This mainly takes place in the QRS complex, in addition to others such as short Q-T and long Q-T waves with high frequencies involved.

**Figure 14:**
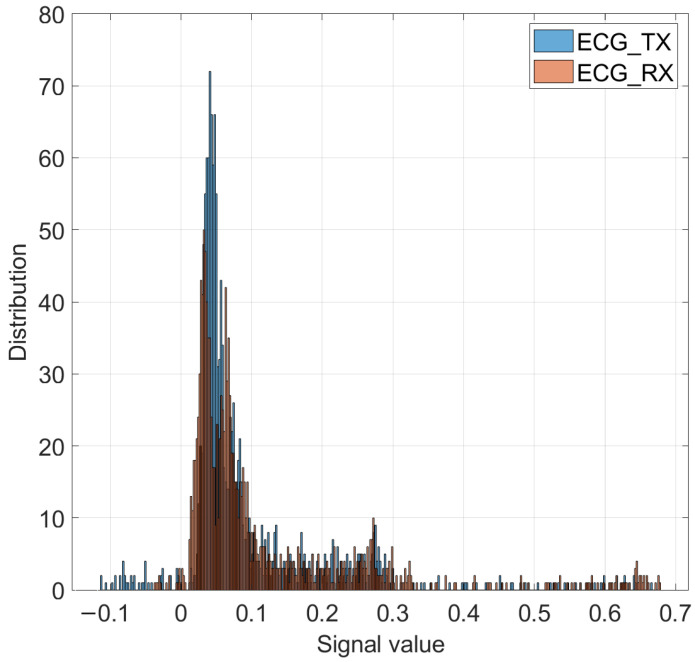
Histogram of transmitted and received ECG signal.

Figure 14 shows the relationship and trend of values throughout the transmission; the distribution of data by voltage range is shown in a relative equivalent compared to the transmitted signal concerning the 4.5% of the received data in the negative region. From 0 to 0.1, there is a distribution of 33.5% of the data; in the voltage ranges of 0.1–0.3, a distribution of 37% is observed, and in the higher-voltage peaks, it corresponds to 25% of the total data. This is the result of the 1402 samples that were utilized in the ECG.

The transmission channel of the AD9361 transceiver provides a wide attenuation range that helps the design optimize the signal-to-noise ratio (SNR). In the transmitter stage, a constant called the scale factor is established during programming, which indicates the power level used within the board with these internal power amplifiers. This scale factor operates from 0 to 1.0, where the higher value indicates that you have taken it to saturation; in this implementation, the preamplifier was operated in the linear region of 0.5 to eliminate unwanted effects at the input of the external power amplifier. For LTE applications with bandwidth less than 10 MHz, there is a Tx SNR of 128 dB/hZ; with this level of linearity, there is a bit error rate of $10^{-4}$, which indicates an error of 0.01%. In this condition, the external power amplifier adds nonlinearities to the system. However, the PA is the primary device in a transmission chain.

## 4. Conclusions

This research demonstrates the effectiveness of chaotic encryption, leveraging inherent randomness and sensitivity to initial conditions to enhance data security. The encryption method relies on synchronizing a master–slave chaotic system, where an ECG signal is encrypted using a master system (multi-scroll chaotic system) and decrypted using a slave system (extended state observer). Lyapunov stability analysis establishes sufficient conditions to ensure error state responses remain uniformly bounded around zero.

The proposed system offers a viable alternative for processing and transmitting ECG signals in telemedicine. It is primarily based on four algorithms: the modulation and encryption of ECG signals, the generation and transmission of LTE signals, the post-processing of received signals, and the decryption of ECG signals.

An FPGA development board was employed to design and integrate an RF transmission platform encompassing digital and power stages. A power amplifier, protected by a directional coupler and designed for base station and LTE applications, enhances power transmission. The experiments utilized the 128-QAM modulation scheme, demonstrating the system’s adaptability to higher schemes up to 1024-QAM.

In this wireless transmission, data at the spectral level and data distribution analysis are compared to guarantee that the correlation of the received signal is ideal so that the health specialist can give an appropriate ECG diagnosis.

The research confirmed the system’s capability to prevent channel overlap during 2.5 GHz transmissions. The 2 GHz band, widely used in North America for RF signal transmission, was employed in the experiments. The precise recovery of signals, including frequencies induced in QRS complexes and QT waves, ensures accuracy for further analysis and classification, showcasing the system’s potential for broader telemedicine applications.

In future work, a classifier system for the self-diagnosis of pathologies can be developed as a means of comparison for the doctor.

## Data Availability

The original contributions presented in the study are included in the article. Further inquiries can be directed to the corresponding author.

## Abbreviations

The following abbreviations are used in this manuscript:

ESO	extended state observer
